# Aquaporin-5–specific heavy chain VDJ knock-in (A5H) mice reveal molecular mimicry–driven initiation and diversification of autoreactive B-cell responses

**DOI:** 10.3389/fimmu.2026.1866158

**Published:** 2026-07-02

**Authors:** Hyunjin Kim, Nayoon Lee, Sabin Acharya, Sungmin Kim, Youngnim Choi

**Affiliations:** 1 Department of Immunology and Molecular Microbiology in Dental Sciences, Seoul National University and Dental Research Institute, Seoul, Republic of Korea; 2 Department of Translational Medicine, College of Medicine, Seoul National University, Seoul, Republic of Korea; 3 Department of Neurology, Seoul National University Hospital, Seoul, Republic of Korea; 4 Department of Neurology, College of Medicine, Seoul National University, Seoul, Republic of Korea

**Keywords:** anergy, aquaporin-5 (AQP5), autoantibody diversification, B-cell development, germinal center responses, molecular mimicry

## Abstract

Molecular mimicry, where microbial antigens resemble self-antigens, is implicated in triggering autoimmune responses, yet the early B-cell events leading to autoantibody production remain unclear. Here, we generated A5H mice, a knock-in model in which a heavy chain binding to the aquaporin-5 (AQP5) “E” epitope (AQP5E) preferentially generates B cells recognizing a microbial mimic peptide (PmE-L). A5H mice exhibited normal B-cell development with a phenotypic reduction in the anergy-associated B-cell compartment and an expanded pool of mimic-reactive B cells. At steady state, A5H mice produced anti-PmE-L antibodies, whereas anti-AQP5E autoantibodies were not detectable by ELISA. Upon immunization with PmE-L, mimic-reactive B cells were recruited into germinal center responses, leading to robust production of anti-AQP5E autoantibodies that cross-reacted with homologous AQPs (AQP4 and AQP1). Furthermore, A5H mice generated anti-AQP5E autoantibodies upon immunization with streptavidin-complexed PmE-L, indicating that A5H-derived mimic-reactive B cells can overcome competition with the immunodominant carrier antigen streptavidin. However, evidence of intramolecular epitope spreading to native AQP5 was not observed. Consistently, tissue deposition of autoantibodies and salivary gland pathology remained minimal. Notably, autoantibody responses were stronger in heterozygous (+/A5H) than homozygous (A5H/A5H) mice, indicating that allelic inclusion enhances autoreactive B-cell activation. These findings establish A5H mice as a valuable model to dissect how molecular mimicry initiates and diversifies autoreactive B-cell responses, advancing understanding of autoimmune disease initiation.

## 1 Introduction

Autoantibodies that recognize self-antigens rather than foreign pathogens are a hallmark of autoimmune diseases and play a central role in their pathogenesis. Although self-reactive B cells can arise during B-cell development, multiple immune tolerance mechanisms—including deletion, anergy, receptor editing, and inhibitory receptor signaling—normally function to restrain these potentially harmful cells (1, 2). However, these protective mechanisms can fail under certain conditions, and molecular mimicry has emerged as an important trigger of autoimmunity. Molecular mimicry occurs when structural or sequence similarities between foreign antigens derived from viruses or bacteria and self-antigens induce cross-reactive immune responses. Classic examples include cross-reactivity between Epstein–Barr virus nuclear antigen-1 (EBNA1) and neuroinflammatory self-antigens in multiple sclerosis and between *Campylobacter jejuni* sialylated lipooligosaccharide (LOS) and human nerve gangliosides in Guillain-Barré syndrome (3, 4). Notably, a molecular mimicry “hotspot” of 47 amino acids within EBNA1 contains multiple cross-reactive epitopes that target proteins such as Glial cell adhesion molecule (GlialCAM), Anoctamin-2, and Alpha-B crystallin, thereby providing a mechanistic link between viral infection, autoantibody production, and the development of autoimmune pathology (3).

Several animal models have been developed to investigate molecular mimicry–induced autoimmunity, including antigen immunization models and experimental autoimmune disease models induced by mimic peptides (5–9). For example, immunization with Epstein–Barr virus EBNA1_AA386–405_ peptide induces both anti-EBNA1 and anti-GlialCAM antibodies and exacerbates experimental autoimmune encephalomyelitis, supporting a role for molecular mimicry in multiple sclerosis (5). Similarly, immunization with *C. jejuni* LOS induces anti-ganglioside GM1 antibodies and peripheral neuropathy resembling Guillain–Barré syndrome (6). We previously developed a mouse model in which germinal center (GC) responses to PmE-L, a peptide derived from *Prevotella melaninogenica* aquaporin, lead to the production of anti–aquaporin-5 (AQP5) autoantibodies associated with Sjögren’s disease (7–9). However, despite these advances, an important unresolved question remains: how do immune responses initially triggered by molecular mimicry develop into autoantibody responses against multiple self-antigens, thereby contributing to the diversification of autoimmune responses?

To address this knowledge gap, we generated an *Igh*-VDJ knock-in (KI) mouse model expressing a B-cell receptor (BCR) with a heavy chain binding to the AQP5 “E” epitope (AQP5E), allowing us to investigate how molecular mimicry shapes B-cell development and the evolution of antigen-specific B cell responses. Specifically, we examined whether AQP5E-reactive B cells are maintained in an anergic state *in vivo* and whether exposure to a microbial mimic can activate these B cells to generate cross-reactive autoantibodies.

## 2 Materials and methods

### 2.1 Mice

All experimental protocols and animal handling procedures were approved by the Seoul National University Animal Care and Use Committee (Seoul, Republic of Korea; No. SNU-21104-2-6). A targeting vector containing a VDJ sequence derived from an AQP5E-binding BCR (9), along with two homology arms, was designed (**Supplementary Figures 1A, B**). Vector construction, gene targeting into the *Igh* locus of C57BL/6J mice, and generation of *Igh*-VDJ KI mice were performed by Cyagen Biosciences Inc. (Taicang, China).

The resulting targeted line, designated A5H, was bred and maintained under specific pathogen-free conditions at the laboratory animal facility of the School of Dentistry, Seoul National University. Genotyping was performed by PCR using the following primer pairs: 5’-CTGATAGGCACCCAAGTACACTA-3’ (forward) and 5’-GTTTTCCAACTCAGTGACTTCATCC-3’ (reverse) for the A5H allele (361 bp); and 5’-GTAAGAATGGCCTCTCCAGGTCTT-3’ (forward) and 5’-CTCCAAAGTCCCTATCCCATCATC-3’ (reverse) for the wild-type (WT) allele (234 bp) (**Supplementary Figure 1C**). The correct integration and frame-integrity of the KI VDJ sequence in the A5H allele were confirmed by Sanger sequencing of the targeted locus, with the resulting sequence alignment shown in **Supplementary Figure 1D**. Male and female mice aged 6–21 weeks were used in experiments.

### 2.2 Antigens and immunization

*P. melaninogenica* KCTC 5457 (Korean Collection for Type Cultures, Daejeon, Korea) was cultured, and lysates were prepared as previously described (9). Peptides used for enzyme-linked immunosorbent assay (ELISA) or tetramer formation were synthesized with N-terminal biotin conjugation, whereas peptides used for flow cytometry were synthesized with fluorescein isothiocyanate (FITC; PmE-L) or Cy5 (AQP5E) conjugation (Peptron, Daejeon, Korea). While PmE-L has both B- and T-cell epitopes, PmE and AQP5E have only a B cell epitope. Streptavidin (SA)-complexed peptide tetramers were prepared as previously described (10). Briefly, SA and biotinylated PmE-L peptides were mixed at a molar ratio of 1:4 and incubated at room temperature for 30 min, followed by overnight incubation at 4 °C. The mixture was then centrifuged through a 50-kDa Amicon filter (Merck), and the tetramer complexes were collected.

For immunization, Dulbecco’s phosphate-buffered saline (DPBS), 100 μg PmE-L peptide, or a tetramer complex containing 100 μg PmE-L peptide was emulsified in incomplete Freund’s adjuvant (IFA) at a 1:1 ratio. Mice were immunized by subcutaneous injection on both sides of the tail base (50 μl per side).

Sample size was estimated using a significance level of α = 0.05 and a power of 80%, based on data obtained from a pilot experiment or the initial set of experiments. Specifically, we used the mean and standard deviation (SD) of AQP5E⁺ cell frequencies in +/+ and +/A5H mice, as well as post-immunization anti-AQP5E autoantibody levels in +/A5H mice.

### 2.3 ELISA

To measure IgG levels against PmE-L, AQP5E, and AQP5 “A” epitope (AQP5A), avidin-coated microplates were incubated with 0.2 μg/well of biotinylated peptides. For detection of anti-SA IgG, microplates were coated with 0.2 μg/well SA (Invitrogen, Carlsbad, CA, USA).

Standard curves were generated by coating serial dilutions of mouse IgG1 (40–0.625 ng/mL; BD Biosciences, Franklin Lakes, NJ, USA) onto each plate. Serum samples were diluted to ensure that optical densities fell within the standard curve range: anti-PmE-L (1:3,000–1:10,000), anti-AQP5E (1:3,000–1:10,000), anti-AQP5A (1:300), and anti-SA (1:10,000). All other procedures were performed as previously described (9).

### 2.4 B cell phenotyping by flow cytometry

Single-cell suspensions were prepared from bone marrow (BM), spleen, and lymph nodes (LN; inguinal, axillary, and brachial, or draining inguinal lymph nodes from immunized mice) by mechanical dissociation followed by filtration through a 40-μm cell strainer.

For antigen-binding assays, cells were incubated with FITC-PmE-L and Cy5-AQP5E at indicated concentrations in RPMI at 37 °C for 1 h. In some experiments, cells were pre-incubated with a ten-fold excess of PmE or AQP5E at 4 °C for 30 min before incubation with labeled peptides for competition. After washing with DPBS, cells were stained with Ghost Dye™ viability dye (Tonbo, San Diego, CA, USA). Fc receptors were blocked using anti-CD16/CD32 antibodies (clone 2.4G2) at 4 °C for 20 min. Cells were then stained with surface antibodies (**Supplementary Table 1**) at 4 °C for 40 min, washed, and analyzed. Data were acquired using LSR Fortessa X-20 (BD Biosciences) and analyzed with FlowJo v10.10.0.

For all samples, live singlets were first gated based on FSC-A versus SSC-A, followed by doublet exclusion using FSC-A versus FSC-H and exclusion of Ghost Dye™–positive dead cells. In BM samples, total B cells were gated as CD19^+^IgM^+^ cells. Immature B cells were subsequently defined as CD93^+^IgD^–^ cells, while mature/recirculating B cells were defined as CD93^–^IgD^+^ cells. In spleen and LN samples, total B cells were gated as CD19^+^ cells among live singlets and further divided into immature (CD93^+^) and mature (CD93^−^) populations. Within the immature population, transitional subsets were identified based on IgM and CD23 expression: T1 (IgM^hi^CD23^−^), T2 (IgM^hi^CD23^+^), and T3 (IgM^lo^CD23^+^) cells. Within the mature population, phenotypically anergy-associated B cells were identified as IgD^+^IgM^lo^ cells on an IgD versus IgM plot. Additionally, marginal zone B (MZB) and follicular B cells within the mature splenic B-cell population were defined based on IgM and CD23 expression as IgM^hi^CD23^−^ and IgM^int/lo^CD23^+^, respectively.

To analyze plasmablasts and GC B cells in the draining LN after immunization, total B cells were gated as TCRβ^–^ cells among live singlets, followed by exclusion of B220^–^ cells. Plasmablasts were defined as CD138^+^ cells among the total B cells. Within the TCRβ^–^B220^+^CD138^–^ population, GC B cells were identified as GL7^+^CD38^–^ cells on a GL7 versus CD38 plot.

### 2.5 Tissue processing and histological analyses

For hematoxylin and eosin (H&E) staining, submandibular salivary glands were fixed in 4% paraformaldehyde at 4 °C for 24 h, embedded in paraffin, sectioned at 4 μm, and stained to assess lymphocytic infiltration.

For immunofluorescence, tissues were fixed in Zinc Fixative (BD Biosciences) overnight at 4 °C, cryoprotected in 10–30% sucrose, and embedded in OCT compound (Leica, Wetzlar, Germany). Frozen sections (10 μm) were fixed in ice-cold acetone for 10 min and blocked with 5% BSA and 10% horse serum in PBS for 1 h. To evaluate IgG binding to AQP5 or AQP4, sections were incubated overnight at 4 °C with rabbit anti-AQP5 (1:200; ATLAS Antibodies, Bromma, Sweden) or rabbit anti-AQP4 (1:100; ATLAS Antibodies), along with mouse sera (1:10). After washing, Alexa Fluor 488–conjugated donkey anti-rabbit IgG and Alexa Fluor 594–conjugated goat anti-mouse IgG (Invitrogen) were applied for 1.5 h. Sections were mounted with antifade medium containing DAPI (Vector Laboratories Inc, Newark, CA, USA) and imaged using an LSM980 confocal microscope (Carl Zeiss, Oberkochen, Germany) at 400× magnification. IgG binding was blindly scored on a 6-point scale (0–5) based on the threshold brightness level (5: detectable at −40%; 4: detectable at -20%; 3: detectable at 0; 2: detectable at +20%; 1: detectable at +40%; 0: undetectable at +40%) at which fluorescence signals remained detectable.

### 2.6 Cell-based assays for detection of AQP4- or AQP1-specific antibodies by flow cytometry

AQP4-specific IgG was measured using a flow cytometry–based cell assay adapted from a previously described method (11). Mouse fibroblast L929 cells stably expressing human AQP4-M23 tagged with EGFP were generated using a lentiviral vector (Twist Bioscience, San Francisco, CA, USA) and maintained under puromycin selection. Parental L929 cells served as negative controls.

HEK293 cells stably expressing human AQP1 were generated by transfection with the pEGFP-N1-AQP1 vector containing full-length human AQP1 cDNA (12), followed by G418 selection.

Cells were cultured under standard conditions and harvested using 0.25% trypsin/EDTA. A total of 1 × 10^6^ cells were incubated with serum samples (1:200 for AQP4; 1:20 for AQP1) for 1 h, followed by staining with secondary antibodies for 30 min: Alexa Fluor 647–conjugated goat anti-mouse IgG (1:500; Jackson ImmunoResearch, West Grove, PA, USA) for AQP4, and Alexa Fluor 594–conjugated goat anti-mouse IgG (1:200; Invitrogen) for AQP1. Cells stained with a recombinant anti-AQP4 antibody generated as described in a previous study (13), and cells stained with rabbit anti-AQP1 IgG (Santa Cruz, Dallas, TX, USA), served as positive controls.

Data were acquired using BD FACS Symphony A5 SE or Fortessa X-20 and analyzed with FlowJo software.

### 2.7 Statistics

All data obtained from the mouse experiments were analyzed using GraphPad Prism (version 9.0; GraphPad Software, San Diego, CA, USA). Normality of data distribution was assessed using the Shapiro-Wilk test. Statistical significance between two groups was determined using an unpaired two-tailed t-test; when the F-test indicated unequal variances (p < 0.05), Welch’s correction was applied. Comparisons involving more than two groups were analyzed by one-way ANOVA followed by Tukey’s multiple comparisons test; when the Brown-Forsythe test indicated unequal variances (p < 0.05), the Brown-Forsythe and Welch ANOVA test was performed instead, with Dunnett’s T3 *post hoc* test. A *p*-value < 0.05 was considered statistically significant. Data are presented as the mean ± SD.

## 3 Results

### 3.1 Generation and B-cell immunophenotyping of A5H mice

From previously identified AQP5E-reactive antibody clones (9), the heavy-chain VDJ sequence of clone 44 was knocked into the mouse *Igh* locus together with leader and J splice donor sequences (**Supplementary Figures 1A, B**). Clone 44 was originally isolated from a phage display antibody library using the microbial mimic peptide PmE, which differs from AQP5E by a single amino acid. Clone 44 contains seven amino acid mutations in the V_H_ derived from the IGHV5-6, IGHD2-3, and IGHJ2 gene segments and four amino acid mutations in the V_L_ region derived from the IGKV14–111 and IGKJ4 gene segments. Recombinant IgG derived from clone 44 binds both PmE and AQP5E, with slightly stronger reactivity toward PmE, while no binding to the unrelated AQP5A epitope is detected (9). Mouse AQP5 shares 95.8% sequence homology with human AQP5 and contains identical sequences at the AQP5A and AQP5E epitopes previously mapped in human AQP5 (**Supplementary Figure 2**). V_H_ replacement was prevented by introducing a TGT-to-TGC silent mutation within the V_H_-internal heptamer (14). In this mouse line (A5H), therefore, all B cells express the clone 44-derived heavy chain, whereas light chains undergo endogenous recombination. Targeting the KI to the *Igh* locus enables somatic hypermutation, affinity maturation, and isotype switching (14).

**Figure 1:**
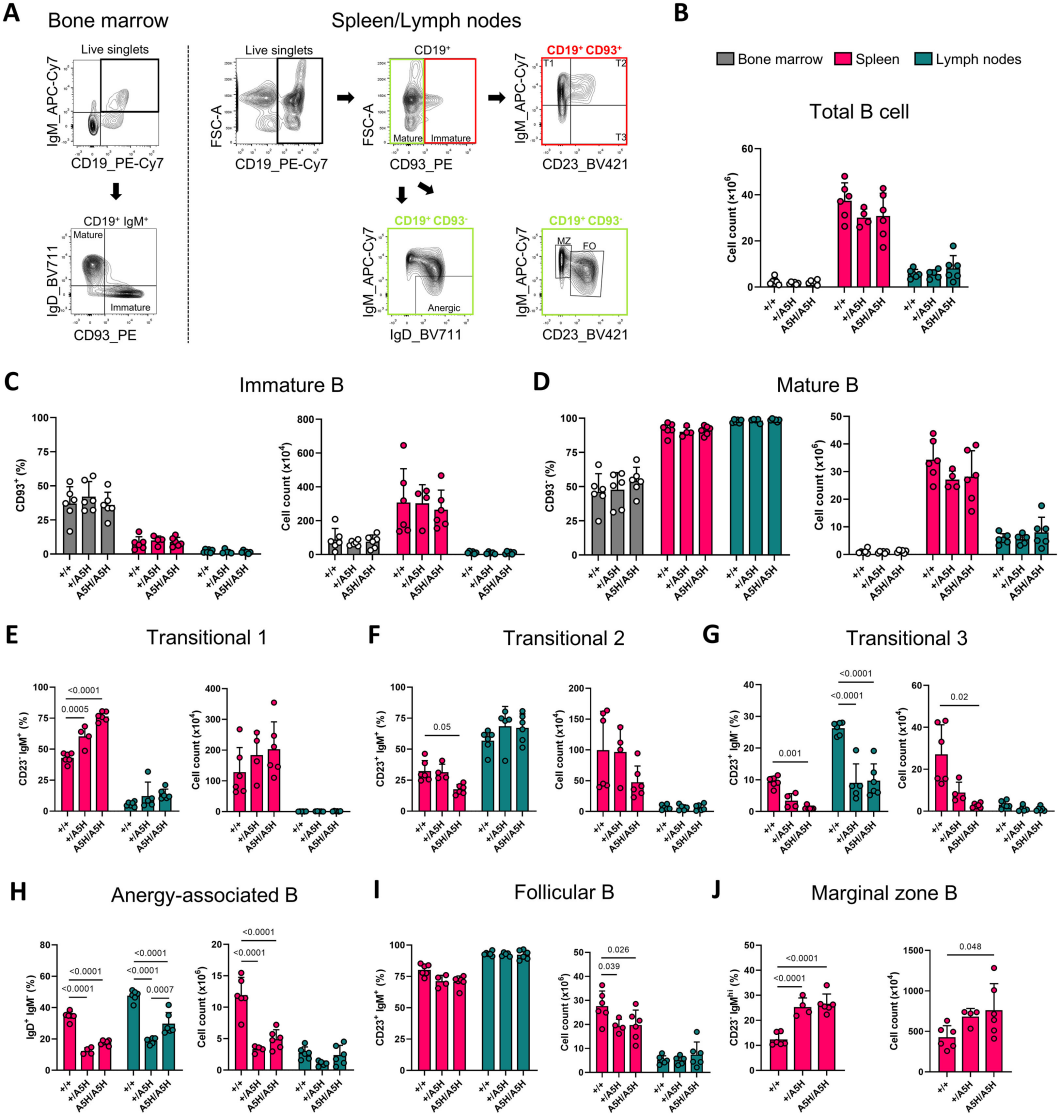
B-cell immunophenotyping of A5H mice. **(A)** Gating strategy for B-cell subset analysis in bone marrow (BM), spleen, and lymph nodes (LN). **(B–D)** Frequencies and absolute numbers of total, immature, and mature B cells in BM, spleen, and LN. **(E–G)** Frequencies and absolute numbers of transitional populations T1, T2, and T3 in A5H mice. **(H–J)** Frequencies and absolute numbers of anergy-associated, follicular, and marginal zone B cells. Combined data from two independent experiments using four male and two female mice (n = 6 per group) are shown as mean ± SD. P values shown above the lines were obtained by one-way ANOVA with Tukey’s *post hoc* test or Brown-Forsythe and Welch ANOVA with Dunnett’s T3 *post hoc* test, depending on the results of the Brown-Forsythe test.

To assess the impact of this autoantibody-derived BCR on B-cell development, we analyzed B-cell compartments in BM, LN, and spleen (**Figure 1A**). Despite the autoreactive nature of the A5H BCR, total B-cell numbers and the proportions of immature and mature B cells were unchanged across tissues, indicating no major central deletion or developmental arrest (**Figures 1B–D**). However, analysis of the transitional B-cell compartment revealed increased T1 and decreased T3 populations (**Figures 1E–G**). In the mature compartment, A5H mice showed reduced frequencies of IgD^+^IgM^lo^ anergy-associated B cells in both the spleen and LN, accompanied by splenic expansion of MZB cells and a reduction of follicular B cells, whereas LN follicular B cells were unaffected (**Figures 1H–J**).

**Figure 2:**
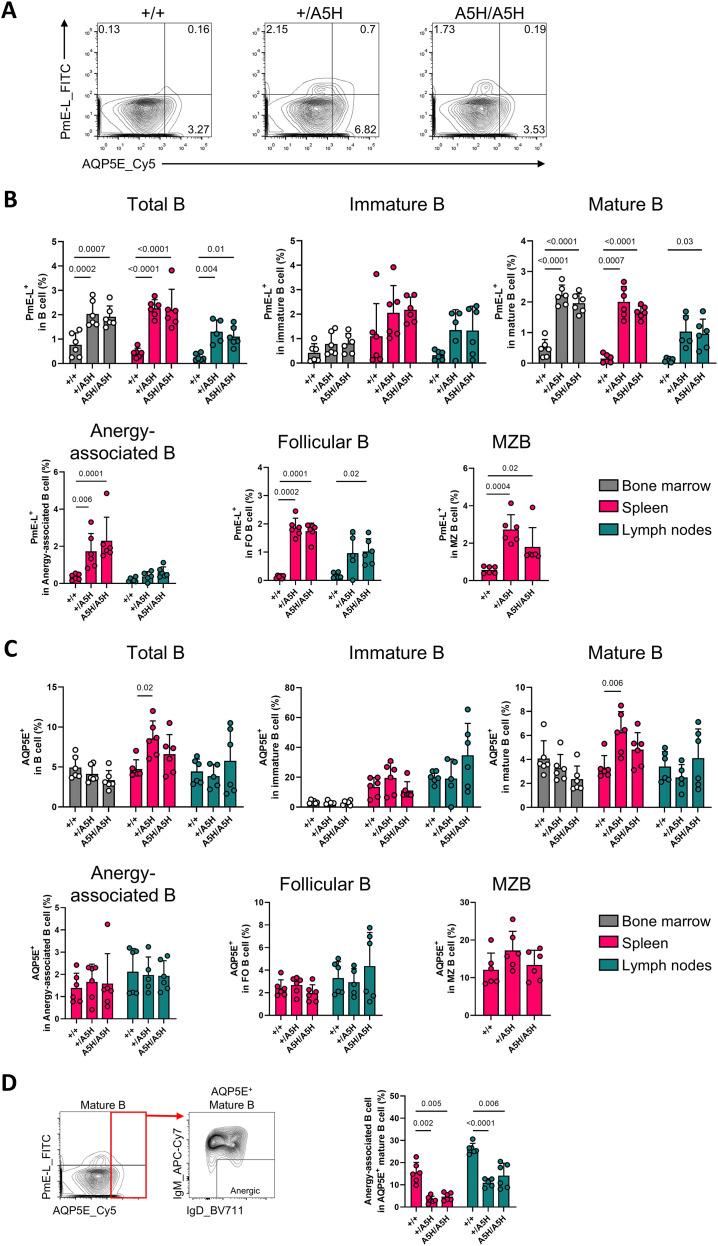
Antigen specificity and anergy of AQP5E-binding B cells. **(A)** Representative contour plots showing AQP5E- and PmE-L–binding cells among splenic total B cells. **(B)** Frequencies of PmE-L–binding cells in each B-cell compartment measured at 100 nM probe concentration. **(C)** Frequencies of AQP5E-binding B cells in each B-cell compartment measured at 100 nM probe concentration. **(D)** Frequencies of anergy-associated B cells among AQP5E-binding mature B cells. Combined data from two independent experiments using four male and two female mice (n = 6 per group) are shown as mean ± SD. P values shown above the lines were obtained by one-way ANOVA with Tukey’s *post hoc* test or Brown-Forsythe and Welch ANOVA with Dunnett’s T3 *post hoc* test, depending on the results of the Brown-Forsythe test.

To assess antigen specificity, we measured binding to AQP5E and its mimic PmE-L using probe concentrations of 100 nM and 10 nM. Under lower-stringency conditions (100 nM), PmE-L–binding B cells were increased in most compartments in both +/A5H and A5H/A5H mice (**Figure 2B**). In contrast, AQP5E-binding B cells were increased only in total and mature B-cell compartments of the +/A5H spleen. Notably, the frequency of AQP5E-binding B cells was substantially higher in immature B and MZB cells than other compartments, even in WT mice (**Figure 2C**), consistent with the known fate of autoreactive B cells (2). Under higher-stringency conditions (10 nM), PmE-L binding was comparable across genotypes, whereas AQP5E-binding cells remained enriched in the +/A5H spleen, particularly within marginal zone and follicular compartments but not among anergy-associated B cells (**Supplementary Figure 3**). The antigen specificity of AQP5E-binding cells in the spleen was further validated by a competition experiment. Competition with excess unlabeled PmE, but not AQP5E, reduced AQP5E-binding B cell frequencies, indicating that the AQP5E-binding B cells detected are predominantly A5H-driven and bind AQP5E through a PmE-cross-reactive specificity with higher affinity for PmE than for AQP5E (**Supplementary Figure 4**). This affinity hierarchy is consistent with the origin of the A5H heavy chain, as Clone 44 was isolated using PmE as bait and shows slightly stronger reactivity toward PmE than AQP5E. We therefore examined the frequency of anergy-associated cells among AQP5E-binding mature B cells. Interestingly, the proportion of anergy-associated cells was reduced in A5H mice in both the spleen and LN (**Figure 2D**).

These results indicate that the A5H heavy chain preferentially generates a pool of mimic-reactive B cells through pairing with endogenous light chains. In contrast, AQP5E-binding B cells are selectively enriched in splenic mature compartments of +/A5H mice, and a substantial proportion retains an IgM^hi^ phenotype, suggesting a relative absence of the IgD^+^IgM^lo^ anergy-associated phenotype in this population.

### 3.2 Antibody and GC responses to immunization with PmE-L in +/A5H mice

**Figure 3:**
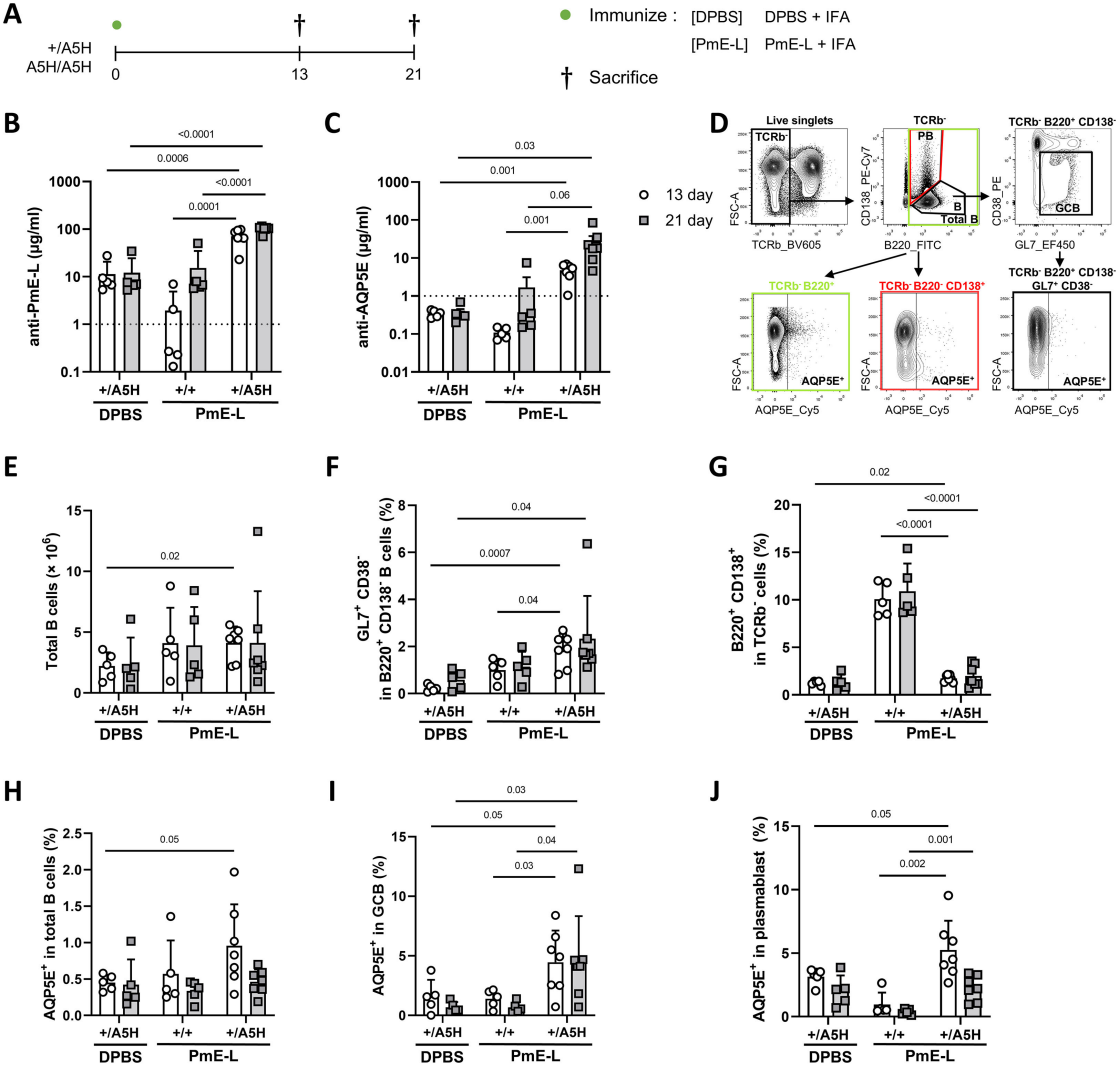
Enhanced antibody and germinal center (GC) responses following PmE-L immunization in +/A5H mice. **(A)** Immunization scheme. +/A5H mice were immunized with PmE-L in IFA and compared with sham-treated +/A5H and PmE-L–immunized WT mice. Sham +/A5H: n = 5; immunized WT: n = 5; immunized +/A5H: n = 7; per group for both time points, all mice 6–14 weeks of age. **(B, C)** Serum antibody responses against PmE-L and AQP5E over time, measured by ELISA using sera diluted 1:3,000. **(D)** Gating strategy for analysis of plasmablasts, total B cells, GC B cells, and AQP5E-binding B cells in draining LN. The AQP5E-binding B-cell frequencies were determined using 10 nM probe concentration. **(E)** Total B-cell numbers in draining LN. **(F)** GC B-cell frequencies. **(G)** Plasmablast frequencies. **(H)** Frequency of AQP5E-binding B cells among total B cells. **(I)** Frequency of AQP5E-binding B cells within GC B cells. **(J)** Frequency of AQP5E-binding B cells within plasmablasts. Combined data from three independent experiments are shown as mean ± SD. P values shown above the lines were obtained by unpaired two-tailed t-test or Welch’s t-test, depending on the results of the F-test for equality of variances.

We next determined if the expanded pool of mimic-reactive B cells enhanced anti-AQP5E autoantibody production compared to WT. +/A5H mice were immunized once with PmE-L in IFA and compared with sham-immunized +/A5H and PmE-L–immunized WT mice (**Figure 3A**).

Sham +/A5H mice produced anti-PmE-L but not anti-AQP5E autoantibodies. Following immunization, both antibody types increased significantly at days 13 and 21. In WT mice, which require at least two immunizations to induce anti-AQP5E antibodies (9, 10), anti-AQP5E responses were detected in only one mouse. In contrast, all +/A5H mice produced anti-AQP5E autoantibodies by day 13, with further increases by day 21 (**Figures 3B, C**).

Flow cytometry of draining LN showed comparable total B-cell numbers and AQP5E-binding frequencies among total B cells across groups. However, GC B-cell frequency and AQP5E-binding within GC B cells were increased in PmE-L–immunized +/A5H mice. Although total plasmablast frequency was lower in +/A5H mice than WT, AQP5E-binding plasmablasts were enriched (**Figures 3D–J**).

These findings suggest that an increased precursor frequency of mimic-reactive B cells facilitates their recruitment into GC responses, promoting efficient production of anti-AQP5E autoantibodies.

### 3.3 Genotype-dependent antibody responses with no sex effect and limited tissue deposition of autoantibodies

**Figure 4:**
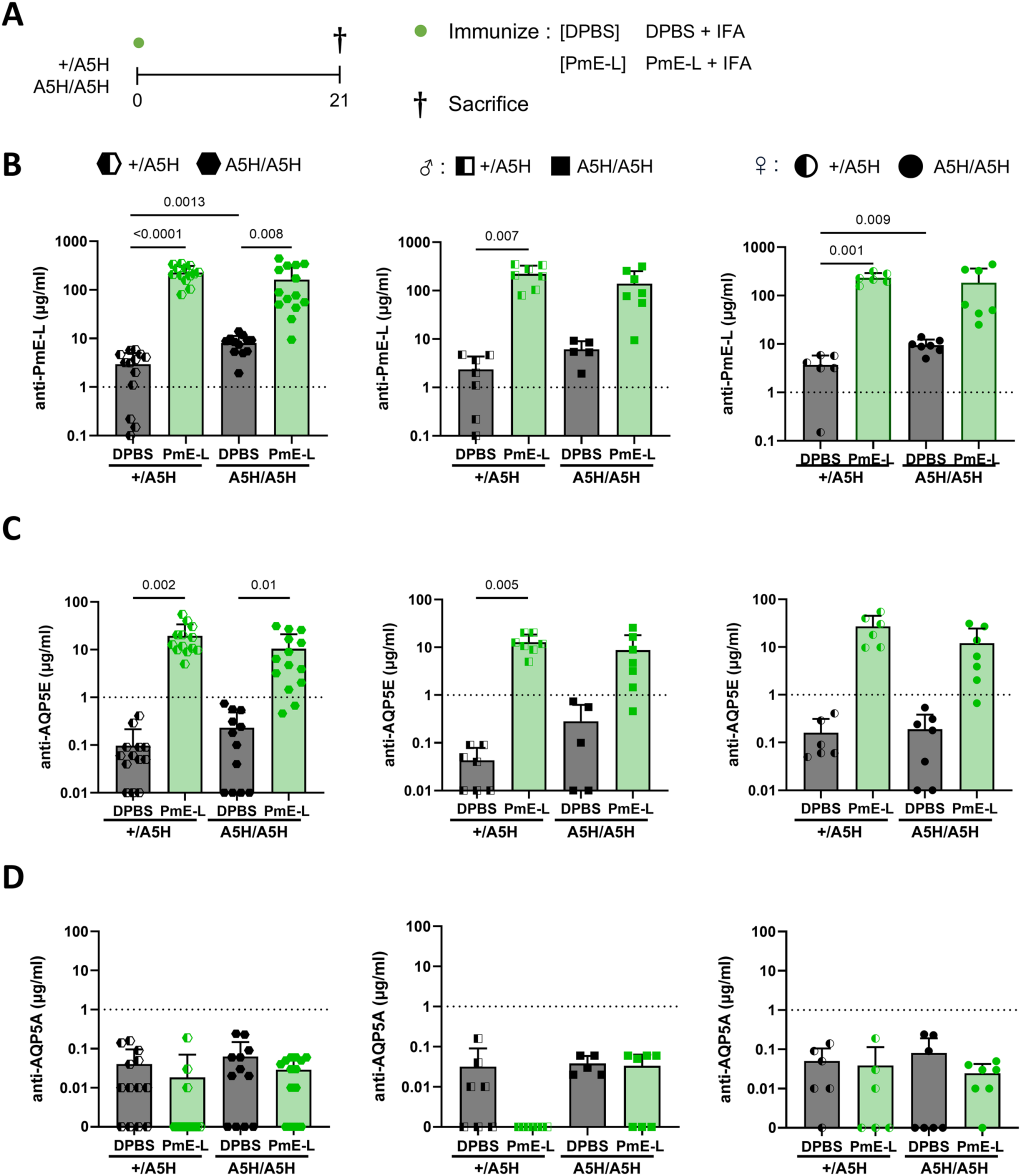
Genotype- and sex-dependent antibody responses. **(A)** Experimental design. +/A5H and A5H/A5H mice were immunized with DPBS or PmE-L in IFA and analyzed on day 21. +/A5H DPBS: n = 13 (7M + 6F, 7–14 weeks old); +/A5H PmE-L: n = 13 (7M + 6F, 7–14 weeks old); A5H/A5H DPBS: n = 11 (5M + 6F, 8–21 weeks old); A5H/A5H PmE-L: n = 14 (7M + 7F, 8–21 weeks old). **(B–D)** Serum antibody levels against PmE-L, AQP5E, and AQP5A, determined by ELISA using sera diluted 1:10,000, 1:3,000, and 1:300, respectively. Combined data from two independent experiments are shown as mean and individual values ± SD. P values shown above the lines were obtained by one-way ANOVA with Tukey’s *post hoc* test or Brown-Forsythe and Welch ANOVA with Dunnett’s T3 *post hoc* test, depending on the results of the Brown-Forsythe test.

We next assessed whether antibody responses and salivary gland pathology differed by genotype or sex. Male and female +/A5H and A5H/A5H mice were immunized with DPBS or PmE-L and analyzed at day 21 (**Figure 4A**).

In sham mice, anti-PmE-L antibodies were readily detectable and were higher in A5H/A5H mice than in +/A5H mice, whereas anti-AQP5E antibodies were not detected in either genotype. Following immunization, both antibody types increased in both genotypes but tended to be lower in A5H/A5H mice: two PmE-L–immunized A5H/A5H mice did not produce anti-AQP5E antibodies. Antibody responses were largely similar between sexes (**Figures 4B, C**).

To determine whether A5H mice generate autoantibodies through epitope spreading, we measured antibodies against the AQP5 “A” epitope, a distinct extracellular epitope of AQP5 that is not present in the PmE-L peptide. Anti-AQP5A autoantibodies were not detected in any mice (**Figure 4D**).

Despite high anti-AQP5E levels, histological analysis showed minimal lymphocytic infiltration, with only one mouse exhibiting a focus score ≥1 (**Supplementary Figures 5A, B**). IgG deposition was detected in only a few mice (**Supplementary Figures 5C, D**), indicating limited tissue accessibility of circulating antibodies.

These results demonstrate that anti-AQP5 autoantibodies are efficiently generated but show limited tissue deposition and minimal pathological consequences, indicating that additional factors are required for glandular pathology.

### 3.4 Cross-reactivity of serum antibodies to other AQPs

**Figure 5:**
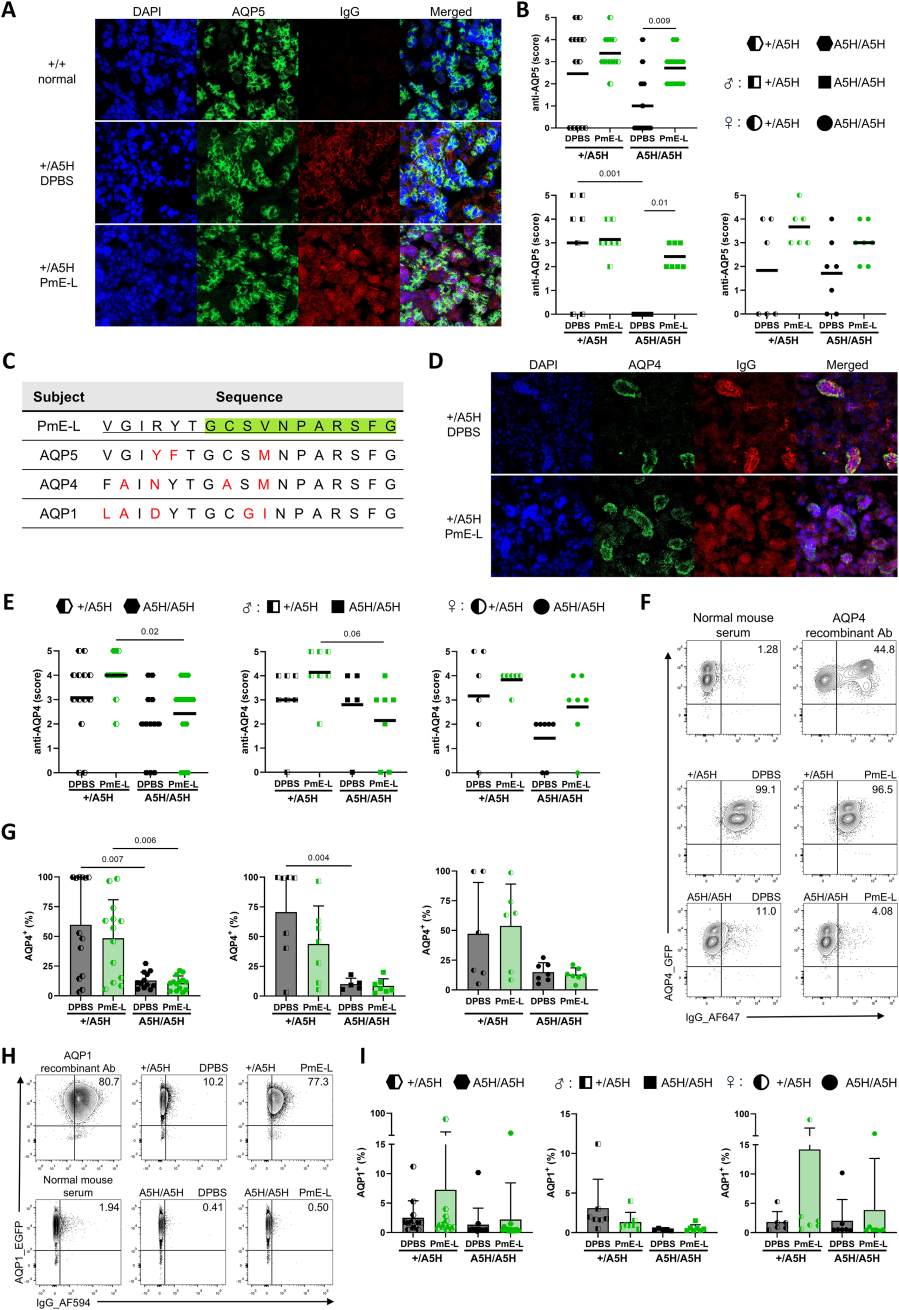
Cross-reactivity of serum antibodies to AQP family members. **(A)** Representative immunofluorescence images of salivary glands showing colocalization of serum IgG with AQP5. **(B)** Quantification of AQP5-binding autoantibody staining intensity. **(C)** Sequence homology among AQP family members in the region corresponding to the B cell epitope AQP5E. The T- and B-cell epitopes in PmE-L are underlined and B-cell epitope is highlighted in green, respectively. Residues differing from PmE-L are shown in red. **(D)** Representative immunofluorescence images showing serum IgG binding to AQP4. **(E)** Quantification of AQP4-binding autoantibody staining intensity. **(F, G)** Cell-based assay quantification of anti-AQP4 antibodies. **(H, I)** Cell-based assay quantification of anti-AQP1 antibodies. Data are shown as mean and individual values. P values shown above the lines were obtained by one-way ANOVA with Tukey’s *post hoc* test or Brown-Forsythe and Welch ANOVA with Dunnett’s T3 *post hoc* test, depending on the results of the Brown-Forsythe test.

To confirm the binding of serum antibodies to AQP5 expressed on acinar cells in the salivary glands, double immunofluorescence staining was performed. Unexpectedly, serum IgG colocalized with AQP5 in 13 of 24 (54%) sham-immunized mice despite the absence of anti-AQP5E antibodies by ELISA, while colocalization was detected in all PmE-L–immunized mice. The intensity of AQP5 colocalization tended to be lower in A5H/A5H mice than in +/A5H mce, particularly in the sham-immunized group (**Figures 5A, B**).

During this analysis, we frequently observed serum IgG binding in ductal structures, suggesting potential cross-reactivity with other AQPs expressed in the salivary glands. Given the high sequence homology among AQP family members—particularly within the region corresponding to the AQP5 “E” epitope (**Figure 5C**)—we next investigated whether these antibodies also recognize AQP4 and AQP1.

Double immunofluorescence staining revealed that serum IgG bound AQP4 in a pattern similar to AQP5. Binding was detectable even in sham-immunized mice and tended to be further enhanced following PmE-L immunization. AQP4-binding intensity was significantly lower in A5H/A5H mice than in +/A5H mice in the PmE-L-immunized group (**Figures 5D, E**). Cell-based assays using AQP4-GFP–expressing cells confirmed significantly higher levels of anti-AQP4 antibodies in +/A5H mice than in A5H/A5H mice in both sham- and immunized conditions (**Figures 5F, G**).

We further assessed reactivity to AQP1 using a cell-based assay. Low-level AQP1 binding was detected in a subset of mice regardless of immunization status, genotype, or sex. Notably, two female mice (one +/A5H and one A5H/A5H) developed high levels of anti-AQP1 antibodies following PmE-L immunization (**Figures 5H, I**).

These results indicate that molecular mimicry can drive the expansion of cross-reactive B-cell responses to multiple homologous self-antigens in this model.

### 3.5 Preferential responses of mimic-reactive B cells under a restricted BCR repertoire

Production of anti-AQP5 autoantibodies typically requires GC–mediated somatic hypermutation (9). We previously showed that incorporation of a highly immunogenic epitope such as SA into the immunizing antigen skews B-cell responses toward extrafollicular pathways, thereby preventing the generation of anti-AQP5 autoantibodies in WT mice (10).

**Figure 6:**
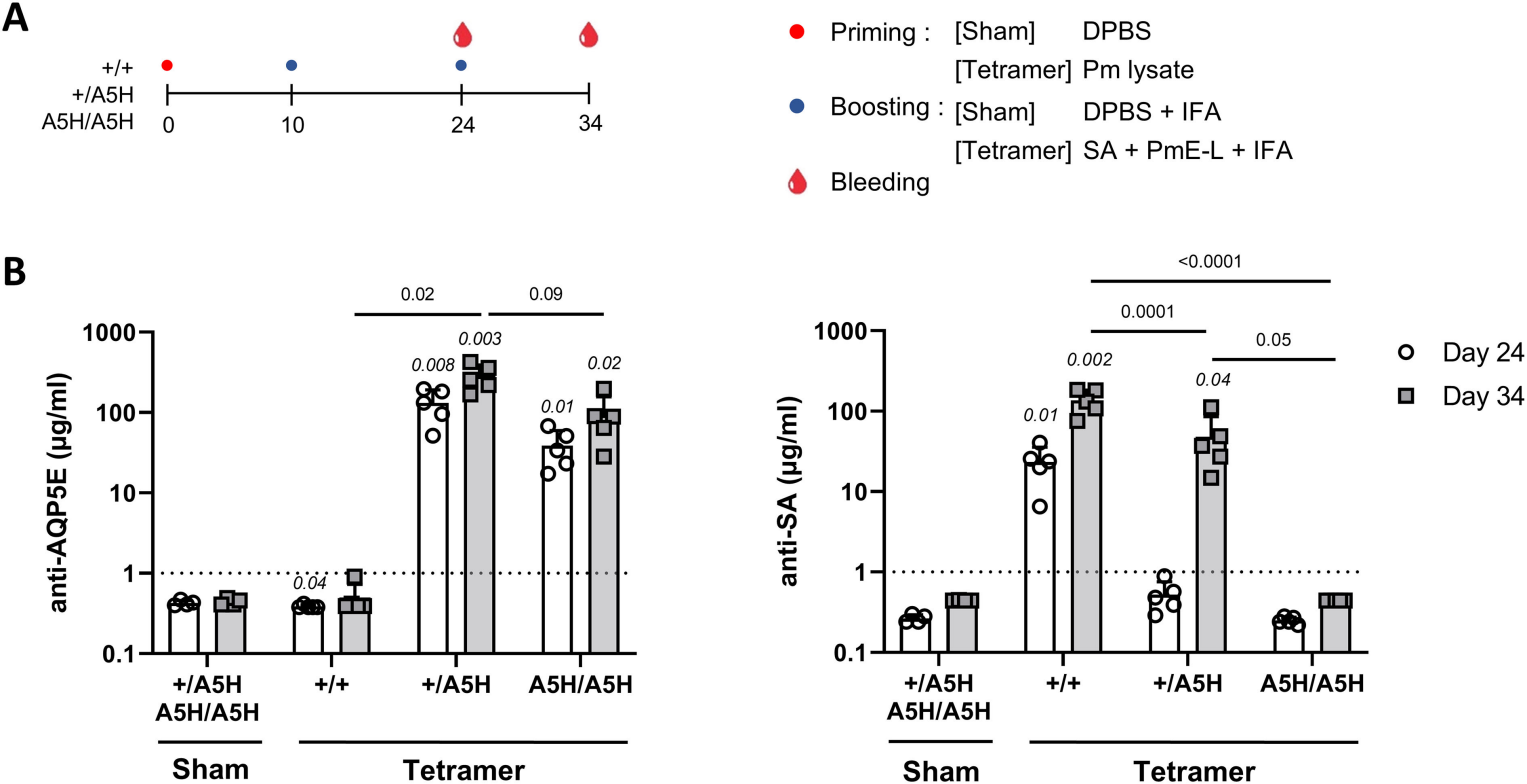
Preferential responses of mimic-reactive B cells in A5H mice. **(A)** Immunization scheme. Mice of different genotypes were primed with *P. melaninogenica* lysate and boosted twice with streptavidin (SA)-complexed PmE-L tetramer. Sham: n = 2 per genotype (4 total); immunized +/+: n = 5; immunized +/A5H: n = 5; immunized A5H/A5H: n = 5; per group for both time points, all mice 6–15 weeks of age. **(B)** Serum antibody responses against AQP5E and SA, determined by ELISA using sera diluted 1:10,000. Data are shown as mean ± SD. Italicized p values were obtained by unpaired two-tailed t-test or Welch’s t-test versus sham-treated mice, depending on the results of the F-test for equality of variances, whereas other p values shown above the lines were obtained by one-way ANOVA with Tukey’s *post hoc* test or Brown-Forsythe and Welch ANOVA with Dunnett’s T3 *post hoc* test, depending on the results of the Brown-Forsythe test.

To determine whether the restricted BCR repertoire in A5H mice alters this outcome, WT, +/A5H, and A5H/A5H mice were primed with *P. melaninogenica* lysate, and subsequently boosted with a SA-complexed PmE-L tetramer twice. Antibody responses were examined at days 24 and 34 (**Figure 6A**). Consistent with previous findings, WT mice failed to produce anti-AQP5E autoantibodies despite robust anti-SA responses. In contrast, both +/A5H and A5H/A5H mice generated substantial levels of anti-AQP5E autoantibodies as early as day 24, although responses were significantly lower in A5H/A5H mice than in +/A5H mice. Notably, anti-SA antibodies were readily induced following repeated exposure to SA in +/A5H mice but were absent in A5H/A5H mice (**Figure 6B**).

These findings suggest that restriction of the BCR repertoire reshapes antigen dominance during immune responses, reducing competition from immunodominant epitopes and allowing mimic-reactive B cells to preferentially engage in GC responses and generate anti-AQP5 autoantibodies.

## 4 Discussion

This study establishes A5H mice as a novel BCR KI model in which B cells preferentially recognize a microbial mimic (PmE-L) of the self-antigen AQP5. Upon activation, these mimic-reactive B cells acquire self-reactivity through GC responses, resulting in the robust production of anti-AQP5E autoantibodies and cross-reactivity with homologous AQPs.

Compared with conventional autoreactive BCR transgenic (Tg) or KI models, A5H mice exhibit several unique features (**Table 1**). In models where B cells intrinsically recognize self-antigens such as HEL, DNA, or insulin, peripheral B-cell numbers are often reduced due to clonal deletion, while many of the remaining cells become functionally silenced through anergy (14–19). In contrast, A5H B cells preferentially recognize a microbial mimic and maintain normal B-cell numbers. Moreover, A5H mice display reduced frequencies of anergy-associated B cells, even among AQP5E-binding cells. This combination of preserved B-cell numbers and reduced anergy-associated phenotype may reflect the lack of AQP5 expression in lymphoid tissues at steady state (10, 20). However, whether the anergic phenotype observed in AQP5E-binding mature B cells is driven by alternative self-antigens, or the restricted BCR repertoire imposed by the fixed A5H heavy chain limits interactions with diverse self-antigens, remains to be directly tested.

**Table 1:** Unique features of A5H mice compared with conventional autoreactive BCR Tg/KI models.

Feature	Conventional autoreactive BCR Tg/KI models	A5H mouse model
Primary antigen recognition	Direct recognition of self-antigen (e.g., HEL, DNA, insulin)	Preferential recognition of microbial mimic (PmE-L)
Clonal deletion	Clonal deletion leading to reduced B-cell numbers when receptor editing or deletion failed (16–18)	Comparable B-cell numbers
Anergy status	Prominent anergy (14, 15, 17–19)	Reduced anergy-associated B-cell compartment
Trigger of autoimmunity	Spontaneous or induced by self-antigens complexed with TLR ligands (17–19)	Mimic antigen administered with adjuvant
Autoantibody diversification	Typically restricted to defined autoantigen(s)	Mimicry drives cross-reactivity across homologous proteins
Model focus	Tolerance mechanisms or autoreactivity	B cell–centric mimicry model

At steady state, A5H mice exhibit baseline antibodies against PmE-L without detectable anti-AQP5E autoantibodies by ELISA. However, the expanded pool of mimic-reactive B cells, together with reduced anergy-associated phenotype, enables efficient recruitment into GC responses upon stimulation with the mimic peptide, resulting in robust production of anti-AQP5E autoantibodies. Furthermore, A5H mice can overcome competition from an immunodominant antigen such as SA. Together, these findings support a model in which molecular mimicry can recruit B cells that preferentially recognize microbial antigens but harbor latent self-reactivity, and subsequent GC responses can broaden their specificity toward self-antigens.

Most autoreactive BCR Tg or KI models have been studied primarily in heterozygous mice, and the functional consequences of homozygous expression have not been systematically examined (14–19). Our study shows that +/A5H mice mount significantly stronger humoral responses than A5H/A5H mice, as evidenced by increased levels of anti-AQP5E, anti-AQP4, and anti-SA antibodies. The enhanced responses in +/A5H mice may reflect allelic inclusion, whereby simultaneous expression of the A5H and endogenous heavy chain alleles generates B cells expressing multiple BCRs with diverse antigen specificities. Such allelic inclusion often allows autoreactive cells to escape deletion or anergy (21, 22). Consistently, AQP5E-binding B cells were increased in the spleen of +/A5H mice, while anergy-associated B-cell frequencies were reduced in LN compared with A5H/A5H mice. In combination with the ability of IFA to establish secondary antigen depots in LN and spleen (23, 24), the reduced anergic compartment and increased pool of AQP5E-binding B cells are consistent with the efficient production of anti-AQP5E and cross-reactive anti-AQP4 autoantibodies observed in +/A5H mice, though causal relationships remain to be established. In contrast, the absence of anti-SA antibody production in A5H/A5H mice might highlight the functional impact of severe BCR repertoire restriction, which needs to be verified through repertoire sequencing.

Autoantibody responses can diversify through intra- and intermolecular epitope spreading (25). In A5H mice, serum antibodies exhibit cross-reactivity across homologous aquaporins (AQP5, AQP4, and AQP1), indicating diversification driven by molecular mimicry—a distinct form of intermolecular epitope spreading. AQP4 differs from AQP5 by only a single amino acid within the region homologous to the AQP5E epitope, and the presence and magnitude of AQP5- and AQP4-binding autoantibodies are significantly associated (*p* < 0.001, Kendall’s Tau-b test). Consistently, a recent study identified cross-reactive T-cell epitopes in AQP5 and AQP4 that overlap with the AQP5E epitope in mice (26). In contrast, although AQP1 differs by only two amino acids in the homologous region, substantial levels of anti-AQP1 autoantibodies were observed only in a subset of mice. This might reflect a combination of stochastic variation in the light chain repertoire among individual mice and differences in antigen accessibility during B-cell development: AQP1 is widely expressed in red blood cells and vascular endothelium, whereas AQP4 is primarily expressed in astrocytes in the central nervous system and is largely absent from lymphoid tissues (27, 28), potentially leading to deletion of AQP1-reactive B cells.

In the peptide-based PmE-L immunization setting, diversification to another AQP5 epitope (AQP5A) was not observed. This suggests that PmE-L immunization does not provide sufficient exposure to native AQP5 to drive intramolecular epitope spreading, and that cross-reactivity to related AQPs can arise prior to broader exposure to the full self-antigen.

Consistent with limited exposure to endogenous AQP5, antibody deposition in tissues and salivary gland pathology were minimal in this model. These findings indicate that, in this model, circulating autoantibodies alone are insufficient to induce Sjögren-like pathology, and that preceding innate inflammation of the salivary gland epithelium is likely required for tissue damage and sustained autoimmunity (29, 30). Accordingly, the lack of clinically relevant tissue pathology represents a major limitation of the current model.

Collectively, the A5H model provides a unique B cell–centric system in which a pre-existing pool of mimic-reactive B cells acquire self-reactivity through GC responses following activation. This feature enables dissection of early events in autoimmunity, including the transition from microbial antigen recognition to self-reactivity, the role of GC reactions in shaping antibody specificity, and the contribution of factors such as antigen accessibility, inflammatory context, and BCR diversity. In addition, the ability to compare heterozygous and homozygous configurations offers a framework to examine how allelic inclusion and repertoire restriction influence tolerance and activation thresholds. Thus, the A5H model complements existing systems by enabling the study of a pathway of autoantibody generation that is challenging to investigate in traditional models, and provides a valuable platform for exploring mechanisms of autoimmune disease initiation and potential therapeutic interventions.

## Data Availability

The original contributions presented in the study are included in the article/**Supplementary Material**. Further inquiries can be directed to the corresponding author.
