# Supplementary material for: Aquaporin-5–specific heavy chain VDJ knock-in (A5H) mice reveal molecular mimicry–driven initiation and diversification of autoreactive B-cell responses
Source: Front Immunol. 2026 Jul 2;17:1866158. doi: 10.3389/fimmu.2026.1866158 (PMC13373061; doi:10.3389/fimmu.2026.1866158)
Supplement: Supplementary file 1 [file DataSheet1.docx]

Supplementary Material

# Supplementary Figures and Tables

## Supplementary Figures
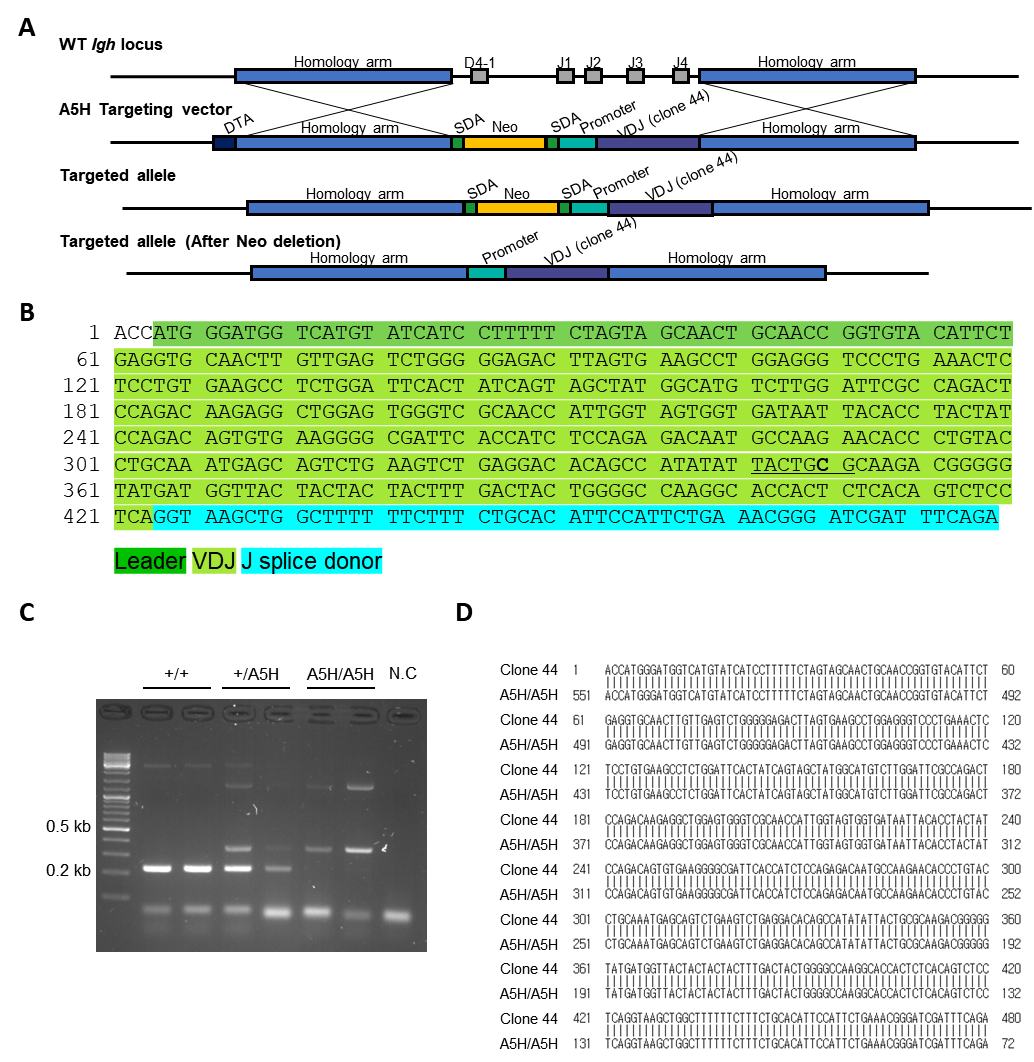


**Supplementary Figure 1.** Gene targeting strategy of A5H. **(A)** The structures of targeting vector and the *Igh* locus. **(B)** The sequence of VDJ in (A) is shown. The V_H_-internal heptamer is underlined, where a TGT-to-TGC silent mutation was introduced. (**C**) Representative agarose gel image of PCR products for genotyping. (**D**) Aligned sequence of the VDJ portion amplified from A5H/A5H mice, confirming successful knock-in of the clone 44-derived heavy chain.


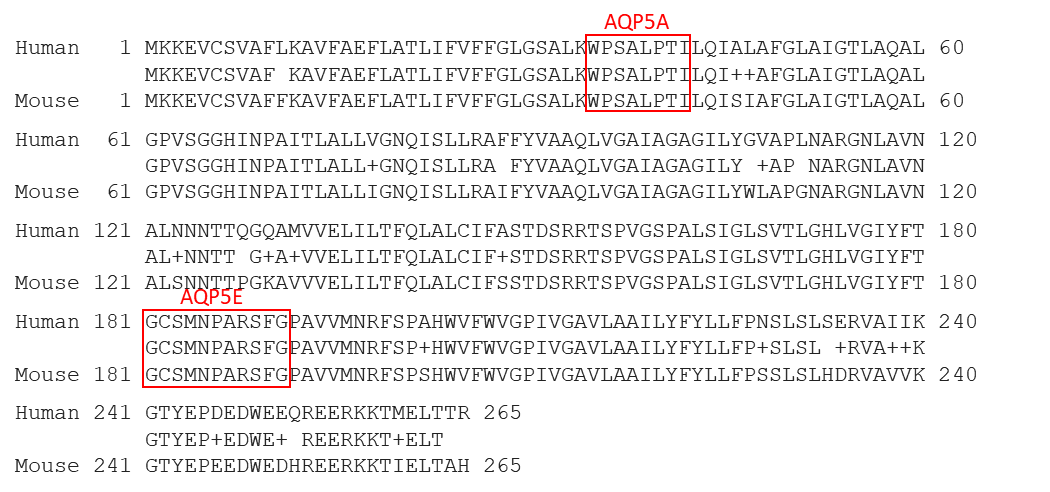


**Supplementary Figure 2.** Aligned sequences of human and mouse AQP5. The AQP5A and AQP5E epitopes are indicated, demonstrating identical sequences between human and mouse at both epitope regions. Overall sequence homology is 95.8%.

**
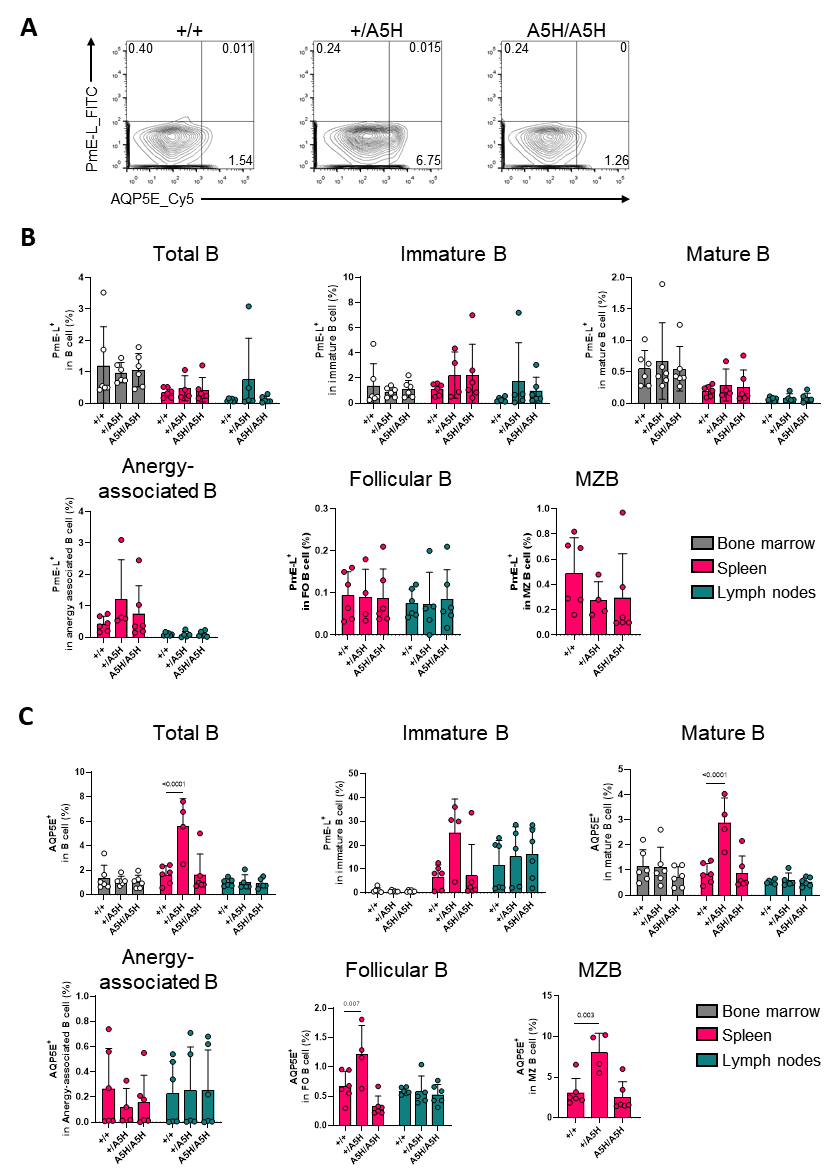
**

**Supplementary Figure 3.** Antigen binding under high-stringency conditions. **(A)** Representative contour plots showing AQP5E- and PmE-L–binding cells among splenic total B cells. **(B)** Frequencies of PmE-L–binding cells in each B-cell compartment measured at 10 nM probe concentration. **(C)** Frequencies of AQP5E-binding B cells in each B-cell compartment measured at 10 nM probe concentration. Combined data from two independent experiments using four male and two female mice (n = 6) per group are shown as mean ± SD. P values shown above the lines were obtained by one-way ANOVA with Tukey’s post hoc test or Brown-Forsythe and Welch ANOVA with Dunnett's T3 post hoc test, depending on the results of the Brown-Forsythe test.

**
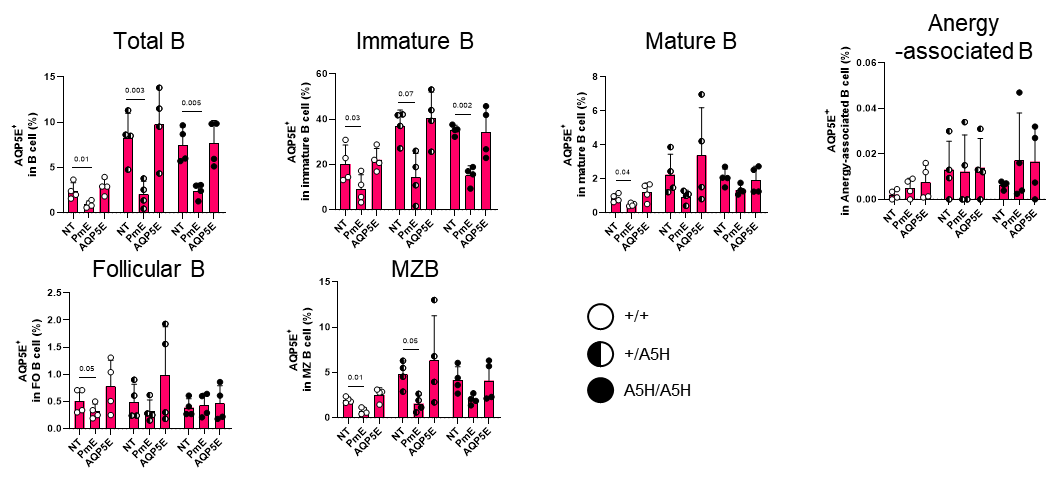
**

**Supplementary Figure 4.** Antigen specificity of AQP5E-binding B cells validated by competition assay. Splenocytes from +/+, +/A5H, and A5H/A5H mice were pre-incubated with a ten-fold excess of unlabeled PmE or AQP5E at 4°C for 30 min before staining with Cy5-AQP5E (10 nM). Frequencies of AQP5E-binding cells among total B, immature B, mature B, anergy-associated B, follicular B, and marginal zone B (MZB) cells are shown. Competition with excess unlabeled PmE, but not AQP5E, reduced AQP5E-binding B cell frequencies across compartments. Competition was not effective in compartments with low baseline AQP5E-binding frequencies, such as anergy-associated B cells. Combined data from two independent experiments (n = 4 per group) are shown as mean ± SD. P values shown above the lines were obtained by unpaired two-tailed t-test or Welch's t-test, depending on the results of the F-test for equality of variances.

**
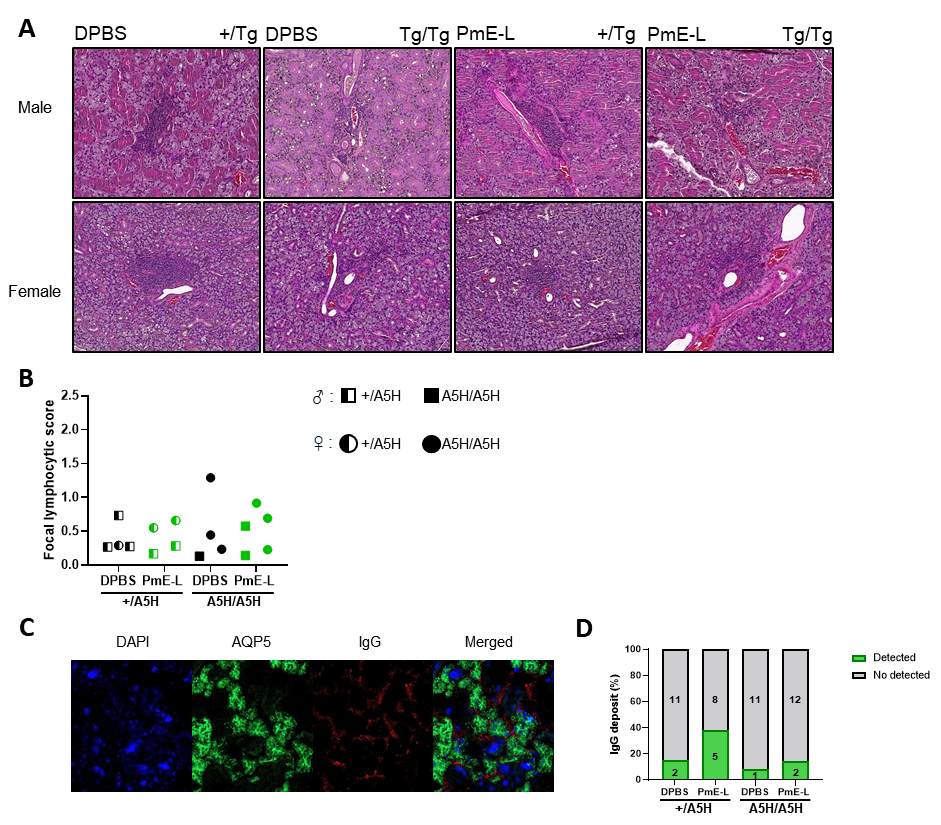
**

**Supplementary Figure 5.** Limited salivary gland pathology and antibody deposition. Submandibular salivary gland tissues from the experiments shown in Figures 4 and 5 were examined for lymphocytic infiltration by H&E staining (**A–B**) and for IgG deposition by immunofluorescence (**C–D**). Representative images (x40) of lymphocytic infiltration are shown in (**A**), and focal lymphocytic sialadenitis scores are shown in (**B**). Representative images of IgG deposition are shown in (**C**), and the frequency of IgG-positive mice in each group is shown in (**D**). Numbers within the bars indicate the number of IgG-positive mice out of the total examined.

## Supplementary Tables

**Supplementary Table 1. List of antibodies used for flow cytometry**

| Antibody | Clone | Source | Cat # |
| --- | --- | --- | --- |
| PE/Cyanine7 anti-mouse CD19 | 6D5 | BioLegend | 115520 |
| PE anti-mouse CD93 | AA4.1 | BioLegend | 136504 |
| Brilliant Violet 421™ anti-mouse CD23 | B3B4 | BioLegend | 101621 |
| APC/Cyanine7 anti-mouse IgM | RMM-1 | BioLegend | 406516 |
| Brilliant Violet 711™ anti-mouse IgD | 11-26c.2a | BioLegend | 405731 |
| PE/Cyanine7 anti-mouse CD138 | 281-2 | BioLegend | 142514 |
| PE anti-mouse CD38 | 90 | BioLegend | 102707 |
| BV605 Hamster Anti-Mouse TCR β Chain | H57-597 | BD Biosciences | 562840 |
| FITC Rat Anti-Mouse CD45R/B220 | RA3-6B2 | BD Biosciences | 01124A |
| GL7 Monoclonal Antibody (GL-7 (GL7)), eFluor™ 450 | GL-7 | Invitrogen | 48-5902-82 |
